# Age of epilepsy onset as modulating factor for naming deficit after epilepsy surgery: a voxel-based lesion-symptom mapping study

**DOI:** 10.1038/s41598-023-40722-4

**Published:** 2023-09-01

**Authors:** Caroline Reindl, Katrin Walther, Anna-Lena Allgäuer, Johannes D. Lang, Tamara M. Welte, Jenny Stritzelberger, Stephanie Gollwitzer, Michael Schwarz, Regina Trollmann, Dominik Madzar, Michael Knott, Arnd Doerfler, Frank Seifert, Karl Rössler, Sebastian Brandner, Stefan Rampp, Stefan Schwab, Hajo M. Hamer

**Affiliations:** 1 Epilepsy Center Department of Neurology, University Hospital Erlangen, Schwabachanlage 6, 91054 Erlangen, Germany; 2 Department of Neurology, University Hospital Erlangen, Erlangen, Germany; 3 Department of Neurosurgery, University Hospital Erlangen, Erlangen, Germany; 4 Department of Neuroradiology, University Hospital Erlangen, Erlangen, Germany; 5 Department of Neuropaediatrics, University Hospital Erlangen, Erlangen, Germany; 6 Department of Neurosurgery, University Hospital Vienna (AKH), Vienna, Austria; 7 Department of Neurosurgery, University Hospital Halle (Saale), Halle, Germany

**Keywords:** Epilepsy, Neurology, Anatomy, Nervous system, Outcomes research, Medical imaging

## Abstract

Age at onset of epilepsy is an important predictor of deterioration in naming ability following epilepsy surgery. In 141 patients with left hemispheric epilepsy and language dominance who received epilepsy surgery at the Epilepsy Centre Erlangen, naming of objects (Boston naming test, BNT) was assessed preoperatively and 6 months postoperatively. Surgical lesions were plotted on postoperative MRI and normalized for statistical analysis using voxel-based lesion-symptom mapping (VBLSM). The correlation between lesion and presence of postoperative naming deterioration was examined varying the considered age range of epilepsy onsets. The VBLSM analysis showed that volumes of cortex areas in the left temporal lobe, which were associated with postoperative decline of naming, increased with each year of later epilepsy onset. In patients with later onset, an increasing left posterior temporobasal area was significantly associated with a postoperative deficit when included in the resection. For late epilepsy onset, the temporomesial expansion also included the left hippocampus. The results underline that early onset of epilepsy is a good prognostic factor for unchanged postoperative naming ability following epilepsy surgery. For later age of epilepsy onset, the extent of the area at risk of postoperative naming deficit at 6 months after surgery included an increasing left temporobasal area which finally also comprised the hippocampus.

## Introduction

Decline in language functions is recognized as potential cognitive deficit following epilepsy surgery and must be considered when planning resections and informing the patient.^1,2^ Naming deficits as assessed by visual confrontation testing, is a commonly reported language morbidity and occurs in about 30% of patients after left temporal resection.^3–5^ Functionally important brain areas for naming have been well studied using a variety of methods. By means of stereoelectroencephalographic mapping of naming, a temporobasal area of the dominant hemisphere can be delineated around the occipitotemporal and collateral sulcus, including the inferior temporal gyrus, the fusiform gyrus and the parahippocampal gyrus.^6,7^ Using fMRI, the middle fusiform gyrus in particular was identified as the lexical semantic hub for naming.^7,8^ The essential character of this basal temporal language area (BTLA) was also demonstrated by studies using postoperative lesion mapping.^9,10^ Hippocampal involvement in naming ability is controversially discussed in the past.^9,11^

Despite the well-documented localisation and organisation of naming function, postoperative naming deterioration is not predictable with certainty as it depends on multiple factors.^12–14^ Using a combination of several predictors, tools like a decision tree or a prognostic nomogram can help to estimate the risk of a postoperative decline in naming performance and correctly classified patients in about 80% of the time.^15,16^ Predictors of postoperative decline in nominal speech are dominant side of resection, better baseline score in naming test, older age at surgery, fewer years of education as well as older age of epilepsy onset.^8,16,17^ The absence of hippocampal sclerosis was also associated with postoperative naming decline in temporal lobe epilepsies.^18^

Some studies found age at epilepsy onset to be the most important factor in determining postoperative naming outcome, with age of onset with the best predictive value varying between studies in a range from 4 to 18 years.^12,14,15,17,19^ However, it is not completely clear how this finding interferes with different patterns of postoperative lesions in the left temporal lobe. Knowing from our previous work that age at epilepsy onset modulates the association of postoperative lesion and naming decline and that this association was seen exclusively with later onset ages^10^, we aimed in this study to investigate this association systematically in more detail. Therefore, we analysed the effect of age of epilepsy onset on the existence or extent of postoperative lesions in the presence or absence of postoperative naming deficits using voxel-based symptom-lesion mapping.

## Methods

### Study cohort

All patients who underwent left hemispheric epilepsy surgery at the Epilepsy Center Erlangen between 1998 and 2020 were identified. All patients underwent non-invasive video-EEG monitoring, 3T or 1.5T MRI and neuropsychological testing as part of the presurgical assessment. This also included a detailed history taking of the individual epilepsy syndrome including age at onset, seizure type and frequency. If clinically advised, additional testing was performed, such as PET scanning, MEG, WADA testing or invasive video-EEG monitoring. Postoperative follow-up six months after surgery usually included repeat MRI and neuropsychological re-testing.

Inclusion criteria for this study comprised availability of high resolution 3D data set of T1 weighted MRI and object naming scores of the Boston naming test (BNT)^20^ preoperatively and 6 months post-operatively. In addition, only patients with left hemispheric dominance for language were included which was based on neuropsychological testing, handedness, and supplementary WADA testing (66 of 141 patients) and fMRI of language function if available. Engel classification was used to score postoperative seizure outcome.^21^ All patients gave written general informed consent for using their clinical data for scientific studies. Our institutional ethical review committee of the University hospital Erlangen approved the conduct of this non-interventional, retrospective study of clinical data and waived a specific ethics application. All analyses were performed in accordance with local guidelines and regulations.

### Pre- and postsurgical neuropsychological assessment

The Boston naming test was used to examine picture naming ability as part of the standard pre- and postoperative neuropsychological evaluation.^20,22^ Patients were requested to name a series of 60 line drawings in succession with increasing difficulty. The correct answer had to be given spontaneously, naming error was noted if the patient's response differed from the target name either semantically or phonologically. Total test performance was calculated as the sum score of all correctly named items. Performance of 48 or more correct answers is considered as a normal test result.^23^ Significant postoperative decline in picture naming function was defined as a decline of ≥ 5 correct answers, corresponding to a reliable change index of 5 points.^24^

### Imaging and lesion delineation

For a detailed description of the methods, we also refer to our previous study.^10^ In brief, magnetic resonance imaging was performed according to a standard epilepsy protocol of the Epilepsy Center Erlangen (3 Tesla, Magnetom Trio or 1.5 Tesla Magnetom Sonata, Siemens Healthcare, Erlangen, Germany) for all patients of the study. T1-weighted 3D datasets with a resolution of 1 × 1 × 1 mm were used for further analysis. Algorithms of SPM 12 (https://www.fil.ion.ucl.ac.uk/spm) were used. As a first step, pre- and postoperative T1 sequences were aligned in reference to the anterior and posterior commissure and coregistered in SPM. In a second step, T1 images were resliced to a 2 × 2 × 2 mm resolution for purposes of manual lesion segmentation. Third, lesions were manually plotted onto the 3D T1 images using MRIcron software (https://www.mricro.com)^25^ and adjusted for a postresection-shift using the preoperative T1 sequence. For this purpose, the lesion delineations were additionally compared with the preoperative 3D T1 images and a manual correction was made for displacement of unresected tissue into the resection cavity. Fourth, lesion maps and postoperative T1 images were normalized with Clinical Toolbox in SPM 12 using enantiomorphic lesion masking and unified normalization-segmentation routines^26,27^ and resulting in normalized images with a resolution of 1 × 1 × 1 mm. The normalized T1 images were used to create an average T1 template and a binary mask for statistical analysis. We, therefore, selected 200 normalized T1 images of high quality from a cohort of 311 epilepsy patients of the Erlangen Epilepsy Center with resections in different regions of the brain^10^ (76 patients of the current sample were included) and calculated a voxel-wise mean using FSL (https://git.fmrib.ox.ac.uk/fsl/); Jenkins 2012).^28^ The average T1 image was than binarized at a defined voxel intensity that resulted in a mask of only brain tissue.

Finally, statistical analyses within the binarized brain mask were performed using these normalised lesion maps with a resolution of 1 × 1 × 1 mm.

### Statistical analysis and voxel-based lesion-symptom mapping

Statistics of demographic and behavioral data were performed using IBM SPSS Statistics 22.0 (http://www.spss.com). Proportions were compared using Pearson’s Chi^2^ test, continuous variables were compared using the Mann–Whitney U test. Two-sided p values of less than 0.05 were considered statistically significant. Variables showing significant differences in the univariate comparison between groups were considered candidate covariates for multivariate analyses using binary logistic regression.

Lesion-symptom mapping was computed with NiiStat (https://www.nitrc.org/projects/niistat). Several VBLSM analyses to calculate correlation of lesion localisation and occurrence of naming decline were calculated, with the difference that patients with certain onset ages were included: The correlation between postoperative lesion and presence of postoperative naming decline was examined voxel-wise varying the age of onset and controlling for total lesion volume in each case. Only voxels with a resection in at least five patients were included in the analysis. The correlation was calculated using the Freedman–Lane test with 10.000 permutations and thresholds were one tailed (*p* < 0.05).^29^ Cluster sizes with a minimum of 100 voxels were visualised as the result. Coordinates of significant clusters were presented in the Montreal Neurological Institute (MNI) space. In addition, we used MNI152 standard-space T1-weighted average structural template image for 3D-visualization in MRIcroGL (https://www.nitrc.org/projects/mricrogl).^25^ The number of voxels in the different brain regions was extracted using masks from the AAL atlas. VBLSM analyses were performed up to an age of onset of 25 years to ensure sufficient statistical power.

### Ethical publication statement

We confirm that we have read the Journal’s position on issues involved in ethical publication and affirm that this report is consistent with those guidelines.

## Results

### Total study cohort

**Figure 1:**
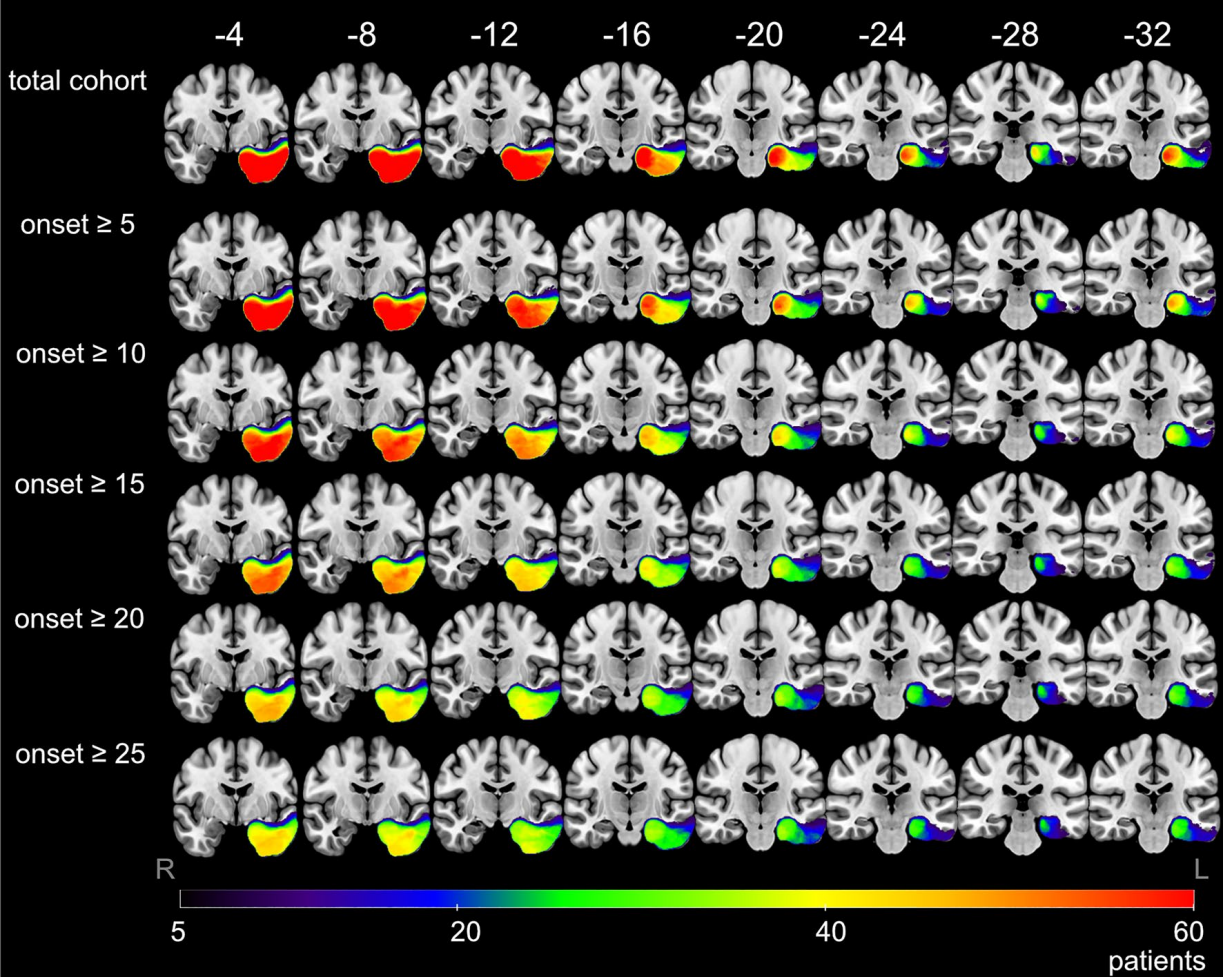
Lesion overlap map showing regions with lesions in at least 5 patients. Total patient group, patients with age of epilepsy onset ≥ 5, 10, 15, 20 and 25 years. Coordinates are presented in MNI space, results are presented on a standard SPM152 template in coronal view. Color bar visualizes number of patients with overlapping resection zones. R: right; L: left.

141 patients with left hemispheric epilepsy and language dominance were included in the study (Table 1). The lesion overlap map for the total patient group shows left hemispheric resections for a minimum overlap of 5 patients (Fig. 1). For detailed neuropsychological results see also the supporting information and previous work.^10^

**Table 1:** Postoperative decline in picture naming in patients with left hemispheric resections.

	No decline of BNT (n = 98)	BNT decline (n = 43)	Uni-variate analysis *p* value	Logistic regression for BNT decline OR (95% CI)	Logistic regression *p* value
Demographics					
Female	48 (49.0)	18 (41.9)	0.437		
Years of schooling	10 (8–12)	9 (8–10)	0.282		
Age at surgery (in years)	35 (25–43)	46 (32–55)	**0.002**	1.03 (0.993–1.073)	0.108
Epilepsy					
Age at onset (in years)	17 (7–27)	28 (19–38)	**0.004**	**1.04 (1.002–1.073)**	**0.040**
Years between epilepsy onset and surgery	12 (4–26)	13 (6–26)	0.548		
Resection type					
Frontal	12 (12.2)	3 (7.0)	0.352		
Temporal lobe	81 (82.7)	40 (93.0)	0.105		
Temporal neocortical	43 (43.9)	13 (30.2)	0.129		
Temporal mesial	38 (38.8)	27 (62.8)	**0.009**	1.83 (0.726–4.632)	0.120
ATL	12 (12.2)	14 (32.6)	**0.004**		
pAH	20 (20.4)	9 (20.9)	0.944		
sAH	6 (6.1)	4 (9.3)	0.500		
Parietal	8 (8.2)	0	0.055		
Occipital	2 (2.0)	0	0.347		
Histopathology					
Hippocampus sclerosis	24 (24.5)	16 (37.2)	0.124		
Cele	4 (4.1)	0	0.181		
Tumor	30 (30.6)	9 (20.9)	0.238		
Cerebral angioma	17 (17.3)	5 (11.6)	0.391		
Malformations of cortical development	12 (12.2)	4 (9.3)	0.613		
Non-lesional	9 (9.2)	10 (23.3)	**0.03**	**3.45 (1.097–10.855)**	**0.034**
Other pathology	2 (2.0)	1 (2.3)	0.914		
BNT preoperative normal	52 (53.1)	27 (62.8)	0.286		
Normalized lesion volume (in ml)	24.5 (7.3–36.7)	33.7 (22.2–43.1)	**0.004**	1.0 (1.0–1.0)	0.073
6 months outcome engel class 1	78 (79.6)	30 (69.8)	0.206		

Values are n (%) or median (interquartile range). Statistically significant values (*p* < 0.05) are expressed in bold. N number, BNT Boston naming test, OR odds ratio, CI confidence interval, ATL anterior temporal lobectomy, pAH resection of temporal pole and amygdalohippocampectomy, sAH selective amygdalohippocampectomy; BNT decline was defined as decline of reliable change index ≥ 5 correct answers.

### Predictors of postoperative naming deficit

In univariate analyses patients with postoperative deterioration of BNT had significantly higher age at epilepsy onset and at the time of surgery (Table 1). Absence of MRI- or histopathologic evidence of an epileptogenic lesion was also significantly associated with postoperative decline in naming, while the etiology of epilepsy was not significantly associated with postoperative naming decline. In addition, more extensive resection volume and temporomesial resection involving the hippocampus were also significantly associated with postoperative worsening of postoperative naming. Considering all univariate significant associated factors, multivariate analysis revealed that only the parameters age at onset and non-lesional epilepsy survived as independent predictors of postoperative naming decline (goodness of fit Nagelkerke 0.27).

### Voxel-based lesion-mapping analyses by age of epilepsy onset

**Figure 2:**
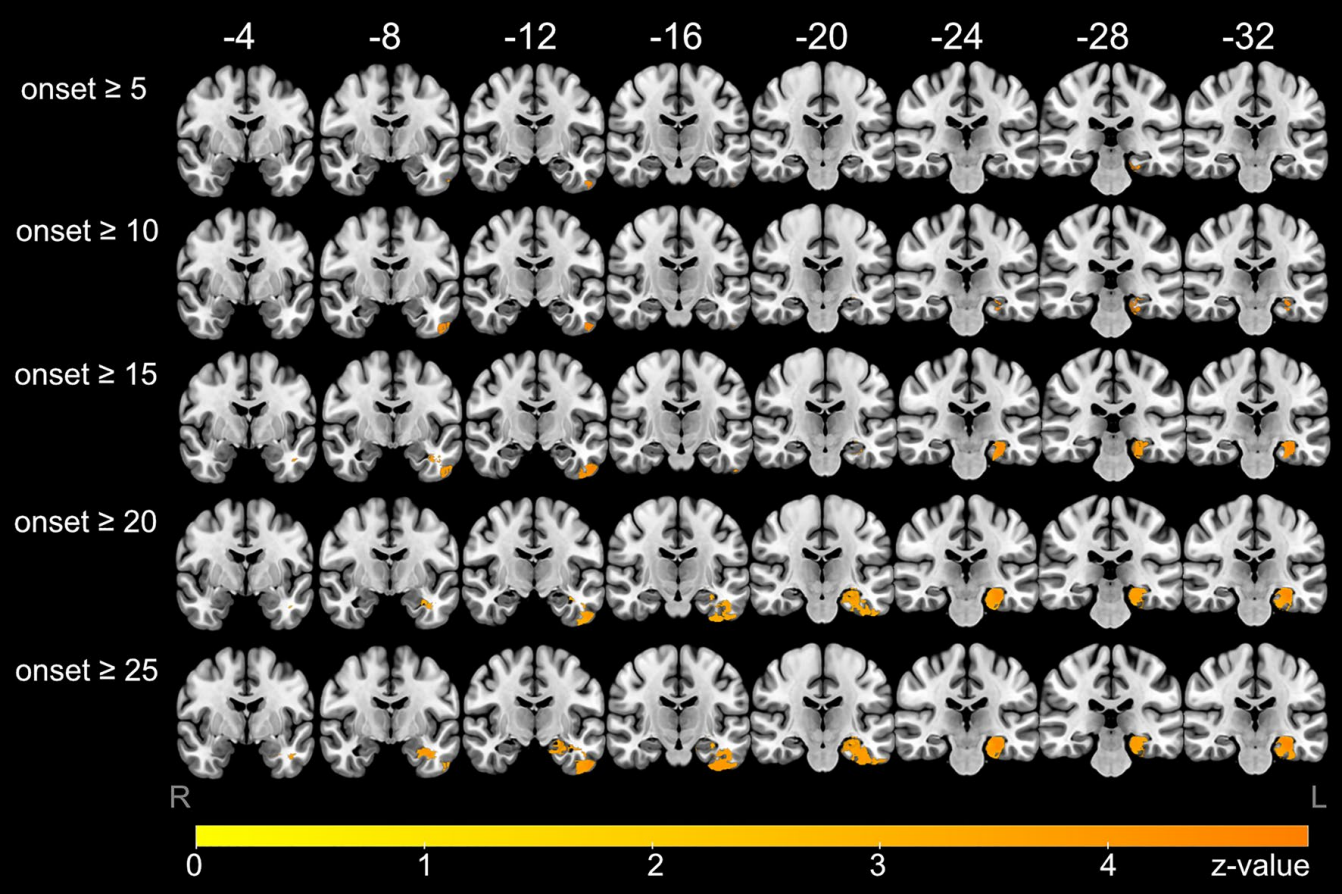
Voxel-based lesion-symptom mapping (VBLSM) showing regions associated with significant postoperative deterioration in picture naming according to age at epilepsy onset. Coordinates are presented in MNI space, results are presented on a standard SPM152 template. Voxels with significant correlation are colour coded according to their *z*-value, *p* < 0.05.

**Figure 3:**
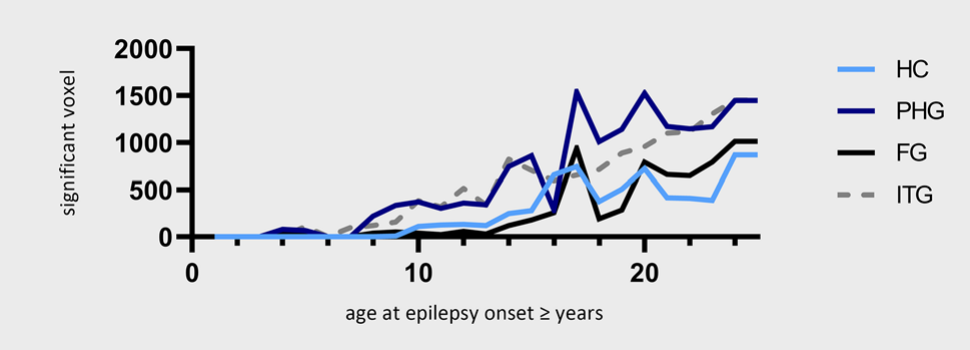
Number of significant voxels of the VBLSM analysis in the respective brain regions in relation to the age of onset of epilepsy. Fusiform gyrus (FG), inferior temporal gyrus (ITG), hippocampus (HC) und parahippocampal gyrus (PHG).

According to age of epilepsy onset, several separate subgroup VBLSM analyses were performed. Up to an age of onset of 5, no cluster > 100 voxels could be delineated (Fig. 3 and supporting information) which predicted postoperative naming decline. In patients with an age of epilepsy onset ≥ 5 years, significant clusters were located in the area of the posterior basal temporal lobe, in the fusiform gyrus and inferior temporal gyrus (Fig. 2). With older age of epilepsy onset ≥ 10 years, there was an increase in cluster size and an expanding temporomesial extent with hippocampal involvement (Figs. 2 and 3). A maximum of the overall significant voxel number was reached in VBLSM of patients with onset of epilepsy ≥ 17 years. This temporobasal to temporomesial cluster extended from the inferior temporal gyrus (ITG), fusiform gyrus (FG) and parahippocampal gyrus (PHG) to the hippocampus (HC) and consisted of 8033 voxels (Fig. 2). In standardized stereotaxic space, the cluster was located in the HC extending from the corpus 38 mm from the temporal pole to the tail 57 mm posterior of the pole, in the PHG 43–58 mm from the temporal pole, in the FG 39–43 mm from the temporal pole and in the ITG 30–43 mm from the temporal pole (MNI coordinates of cluster localization: HC − 24, − 11, − 22 to − 23, − 30, − 13; PHG − 29, − 19, − 25 to − 30, − 31, − 16; FG − 36, − 15, − 40 to − 44, − 21, − 33; ITG − 52, − 5, − 37 to − 50, − 20, − 35).

## Discussion

### Main findings of our study

This voxel-based lesion-symptom mapping study investigated the effect of age at epilepsy onset as predictive factors for postoperative naming deficit on resection areas at risk. Rather than assuming a specific cut-off value for the occurrence of postoperative naming decline, we examined the association of the age of onset as a continuum. Our data confirm higher age at seizure onset as an independent predictor of postoperative naming deficit after left temporal resections. This study extends prior literature by measuring and visualizing the correlation of the area essential for naming function and age of onset. With later epilepsy onset (> 5 years of age), an area of the brain essential for naming function was more likely to be present. With increasing age at epilepsy onset, this area increased and finally included temporomesial areas, like HC and PHG, in addition to temporobasal areas, like FG and ITG.

### Early age at epilepsy onset as protective factor for postoperative naming deficit

Early onset of epilepsy was a good prognostic factor to avoid postoperative naming decline. Better neurocognitive outcome in terms of naming function for early-onset epilepsies versus late-onset epilepsies is well known in literature.^12,14,19^ So far, its anatomical foundation was not well explored. Our study could show that resections in the area of the BTLA lead to a decline in naming only with a higher onset age and no such region in the left temporal lobe could be identified with an epilepsy onset age of < 5 years. We, therefore, hypothesize that early epileptogenic activity in the left temporal lobe occur during a time when neuroplasticity is so great that language networks can be (re-)arranged on an individual level and can be shifted away from the epileptogenic zone leading to atypical language representation and avoiding postoperative naming deficits when the epileptogenic zone is removed. Atypical speech organization is common in epilepsy patients, with a seizure onset at a young age increasing the likelihood of reorganization.^6,30,31^

Intra-hemispheric language reorganization was detected by a precedent study using invasive EEG mapping with the finding of atypically localized naming sites in the anterior temporal lobe (15–35 mm from the temporal tip).^32^ Individual factors such as like organization of language on a regional level, including activation in the dominant FG during picture naming, prognosis postsurgical speech dysfunction and naming decline.^8,33^ Direct cortical stimulation in the middle FG was used to delineate an area in which isolated picture naming was impaired, while sentence repetition and motor function remained intact.^7^ A functional MRI study depicted a significantly higher activation of the left frontal region besides activation of ITG in patients with early epilepsy onset during a visual language task as evidence of intra-hemispheric reorganization.^34^ Better naming function has been shown to be correlated with higher activation of left posterior temporal lobe and stronger functional connectivity within the language network.^35^ Patients with temporal lobe epilepsy showed weaker correlation of functional connectivity than controls.^36^ Estimation of the reorganization pattern using functional MRI at resting state revealed overall reduction of functional connectivity of the language-and-memory-network in patients with mesial temporal lobe epilepsy, while an increased functional connectivity from or to limbic regions was described.^37^

### Later age at epilepsy onset as predictor for postoperative naming deficit

Previous studies reported cut-off values on the basis of behavioral data for an age of onset, after which the risk of a postoperative naming deficit increases, in a range from 4 to 18 years.^15,17,38^ In our data set, we used the age of onset as a continuum to analyse effects of neuronal plasticity.

Our visualization of the area essential for visual naming showed a clear age-at-onset-dependency with maximum of extension from a temporobasal to temporomesial area, including ITG, FG and PHG to HC in late adolescence. Beginning in patients with an age of epilepsy onset ≥ 5 years, we were first able to locate an essential but still small area in the posterior basal temporal lobe, in the fusiform gyrus and inferior temporal gyrus the resection of which induced postoperative naming deficits. With older age of epilepsy onset ≥ 10 years, there was an increase in size including hippocampal involvement. A maximum of the overall significant voxel number was reached in patients with onset of epilepsy ≥ 17 years. The area of neuroplasticity gradually declines over years rendering the brain more and more ineffective to (re-)arrange already established language networks in the left temporal lobe during late childhood and adolescence.

The involvement of the hippocampus in visual naming has long been controversial. Yet, growing evidence suggests a link between hippocampal integrity and visual naming. Studies have found increased hippocampal activity during naming. 1H magnetic resonance spectroscopy demonstrated significant correlations between hippocampal metabolism and naming performance.^39^ Functional MRI showed a correlation for hippocampal activation with visual confrontation naming.^40^ In addition, stereo-encephalographic recording of the left hippocampus via multi-lead depth-electrodes showed high frequency gamma activity being significantly associated with picture naming performance.^11^

Furthermore, the presence of non-lesional epilepsy was an independent predictor of postoperative naming deficits in our data. While previous studies have suggested that patients without hippocampal sclerosis are at higher risk of deterioration in postoperative naming ability, non-lesional epilepsy accompanied by a later age of first manifestation appears to be associated with a higher risk of deterioration in postoperative naming ability overall.^41^

### Strengths and limitations of our study

The method of VBLSM focuses on identifying brain regions that are essentially related to the behavior of interest, representing a strength of our study. This contrasts with functional neuroimaging methods such as fMRI, which identify a more expansive network of regions that correlate with performance on a given task but may not be causally and critically related. In this way, results from lesion analysis techniques such as VBLSM and functional neuroimaging results are complementary.

There are limitations to our work due to its monocentric, retrospective design. According to the age of onset, VBLSM analysis was performed for each subgroup, resulting in a decreasing number of patients with increasing age of onset, with a sample size of 141 in the total cohort decreasing to 58 patients with an epilepsy onset after the age of 25. Precedent studies demonstrated a minimum sample size for reliable VBLSM results of at least 50 patients.^42^ Therefore, no clear conclusions can be drawn for an epilepsy onset age after the age of 25 and we only included results up to an age of onset of 25 years to maintain sufficient statistical power.

In our study, we also based the determination of the minimum sample size on sufficient coverage in terms of lesion overlap. Statistical analyses were only performed in regions with a minimum of five lesions overlapped and eloquent areas tended to be avoided for clinical reasons.

A WADA test or fMRI for a reliable determination of language dominance was performed in in patients with inconsistent findings regarding speech and memory representation and not in all patients. Therefore, it cannot be excluded that a small number of patients with atypical representation were included after all.

## Conclusions

The results of our study show that age of epilepsy onset is an important predictor of the occurrence of postoperative naming deficit by interacting with the location and extent of the area critical for naming function. Later age of onset was associated with greater and further temporomesial extent of brain areas at risk of naming decline when resected. Our results support the assumption that neuroplasticity gradually declines over years rendering the brain more and more ineffective to shift language networks in the left temporal lobe away from the epileptogenic zone during late childhood and adolescence.

### Supplementary Information

Supplementary Information.

## Data Availability

The datasets used and/or analysed during the current study are available from the corresponding author on reasonable request.

## Abbreviations

BNT: Boston naming test
BTLA: Basal temporal language area
CI: Confidence interval
EEG: Electroencephalography
fMRI: Functional magnetic resonance imaging
FG: Fusiform gyrus
ITG: Inferior temporal gyrus
IQR: Interquartile range
OR: Odds ratio
SD: Standard deviation
VBLSM: Voxel-based lesion-symptom mapping
