# Supplementary material for: Age of epilepsy onset as modulating factor for naming deficit after epilepsy surgery: a voxel-based lesion-symptom mapping study
Source: Sci Rep. 2023 Sep 1;13:14395. doi: 10.1038/s41598-023-40722-4 (PMC10474263; doi:10.1038/s41598-023-40722-4)
Supplement: Supplementary file 1 — Supplementary Information. [file 41598_2023_40722_MOESM1_ESM.docx]

**SUPPORTING INFORMATION**

**Table 1:** Number of significant voxels of the VBLSM analysis in the respective brain regions in relation to the age of onset of epilepsy

|  | HC | PHG | FG | ITG | MTG | Number of patients in analysis |
| --- | --- | --- | --- | --- | --- | --- |
| Age at onset ≥ 1 | - | - | - | - | - | 138 |
| Age at onset ≥ 2 | - | - | - | - | - | 135 |
| Age at onset ≥ 3 | - | - | - | - | - | 132 |
| Age at onset ≥ 4 | - | 79 | 29 | - | - | 127 |
| Age at onset ≥ 5 | - | 68 | 25 | 109 | - | 125 |
| Age at onset ≥ 6 | - | - | - | - | - | 121 |
| Age at onset ≥ 7 | - | - | - | 98 | - | 117 |
| Age at onset ≥ 8 | - | 218 | 38 | 119 | - | 112 |
| Age at onset ≥ 9 | 8 | 334 | 51 | 157 | - | 111 |
| Age at onset ≥ 10 | 109 | 371 | 39 | 384 | - | 107 |
| Age at onset ≥ 11 | 125 | 304 | 23 | 319 | - | 106 |
| Age at onset ≥ 12 | 133 | 358 | 59 | 514 | - | 105 |
| Age at onset ≥ 13 | 120 | 340 | 29 | 339 | - | 102 |
| Age at onset ≥ 14 | 248 | 744 | 120 | 823 | - | 99 |
| Age at onset ≥ 15 | 278 | 861 | 179 | 706 | - | 94 |
| Age at onset ≥ 16 | 658 | 289 | 257 | 597 | - | 92 |
| Age at onset ≥ 17 | 746 | 1538 | 938 | 2486 | 52 | 85 |
| Age at onset ≥ 18 | 376 | 1010 | 192 | 715 | 5 | 79 |
| Age at onset ≥ 19 | 504 | 1139 | 286 | 889 | 5 | 77 |
| Age at onset ≥ 20 | 722 | 1527 | 792 | 956 | 5 | 71 |
| Age at onset ≥ 21 | 415 | 1171 | 661 | 1099 | 5 | 67 |
| Age at onset ≥ 22 | 410 | 1145 | 650 | 1114 | 5 | 64 |
| Age at onset ≥ 23 | 388 | 1167 | 791 | 1306 | 5 | 61 |
| Age at onset ≥ 24 | 869 | 1448 | 1011 | 1452 | 20 | 58 |
| Age at onset ≥ 25 | 869 | 1448 | 1011 | 1452 | 20 | 58 |

**Table 2:** Neuropsychological results of naming, semantic fluency, verbal memory and figural memory in our cohort

| **Neuropsychological testing** | Presurgery  mean (SD) | Postsurgery  mean (SD) | Change between pre and post  mean (SD) |
| --- | --- | --- | --- |
| BNT (correct items) | 47.3 (8.6) | 44.5 (9.4) | -2.80 (6.5) |
| Semantic fluency (z-score) | -1.08 (1.36) | -1.14 (1.21) | -0.06 (1.01) |
| Verbal memory (z-score) | -0.81 (0.99) | -0.75 (1.26) | 0.50 (1.03) |
| Figural memory (z-score) | -0,50 (1.15) | -0.27 (1.20) | 0.23 (0.86) |

Abbreviations: BNT Boston naming test, SD standard deviation.

Annotations: Semantic fluency (Regensburger Wortflüssigkeitstest, Aschenbrenner et al. 2001): animals generated in 2 minutes, verbal memory (Berliner Amnesie Test, Metzler et al. 2010): free recall of a learning list of 20 words after 3 minutes of memorization, figural memory (Berliner Amnesie Test, Metzler et al. 2010): immediate reproduction of 10 figures each presented for 5 seconds

**Table 3:** Correlations between Boston naming test (BNT) and cognitive measures of semantic fluency, verbal and figural memory

|  | BNT | | | | | | | |
| --- | --- | --- | --- | --- | --- | --- | --- | --- |
|  | presurgery | |  | postsurgery | |  | Change pre-post | |
|  | r | p |  | r | p |  | r | p |
| Semantic fluency (N = 110) |  |  |  |  |  |  |  |  |
| presurgery | .48 | *** |  | .42 | *** |  | .01 |  |
| postsurgery | .45 | *** |  | .56 | *** |  | .26 | * |
| change between pre - post | -.11 |  |  | .11 |  |  | .30 | * |
|  |  |  |  |  |  |  |  |  |
| Verbal Memory (N = 138) |  |  |  |  |  |  |  |  |
| presurgery | .23 |  |  | .31 | *** |  | .14 |  |
| postsurgery | .15 |  |  | .34 | *** |  | .29 | *** |
| change between pre - post | -.03 |  |  | .13 |  |  | .22 |  |
|  |  |  |  |  |  |  |  |  |
| Figural Memory (N = 127) |  |  |  |  |  |  |  |  |
| presurgery | .11 |  |  | .21 |  |  | .15 |  |
| postsurgery | .14 |  |  | .16 |  |  | .04 |  |
| change between pre - post | .05 |  |  | -.06 |  |  | -.14 |  |

*** p > .001, ** p < .01, * p < .05; Bonferroni-Holms-correction for multiple comparisons, Abbreviations: N number, r Pearson correlation coefficient, p p-value

**References**

Aschenbrenner S, Tucha O, Lange KW. RWT Regensburger Wortflüssigkeits-Test. (Manual). Göttingen: Hogrefe; 2001.

Metzler P, Vohage J, Rösler P. BAT Berliner Amnestie Test. Zur Diagnostik von anterograden mnestischen Störungen. Göttingen: Hogrefe; 2010.
